# Clinical Presentation of Hearing Loss After Contact with a Fish: Case Report

**DOI:** 10.3390/jcm14228010

**Published:** 2025-11-12

**Authors:** Nina Rubicz, Harald Meimberg, Christina Rupprecht, Nikolaus Poier-Fabian, Ulla Folger-Buchegger, Paul Martin Zwittag

**Affiliations:** 1 Department of Otorhinolaryngology, Head and Neck Surgery, Kepler University Hospital GmbH, Krankenhausstrasse 9, 4020 Linz, Austria; 2 Medical Faculty, Johannes Kepler University Linz, Altenbergerstrasse 69, 4040 Linz, Austria; 3 Department of Ecosystem Management, Climate and Biodiversity, Institute of Integrative Nature Conservation Research, BOKU University, Gregor Mendel Str. 33, 1180 Vienna, Austria; 4 ENT Department, Pyhrn-Eisenwurzen Klinikum Steyr, Sierninger Straße 170, 4400 Steyr, Austria

**Keywords:** foreign body, ossicular trauma, inner ear injury, hearing loss, perilymphatic fistula, oval window, case report, vertigo, pregnant women

## Abstract

**Background:** A 33-year-old pregnant woman experienced ear trauma from contact with a fish while swimming. Afterwards, the woman presented with vertigo and hearing loss. **Methods:** Clinical examination showed a foreign body (FB) in the middle and inner ear, which was removed surgically under general anesthesia. Postoperative care included antibiotics; the FB was sent to the lab for analysis. **Results:** Although vertigo resolved after surgical intervention, the woman continued to experience hearing loss and finally experienced deafness. Patient had an unusual mechanism of inner ear trauma. The FB was identified as part of a fish and determined to originate from a fish species of the genus *Hemiramphus* which is listed as harmless to humans in FishBase. **Conclusions:** As severe penetration of an FB into the middle and inner ear can lead to serious complications, removal of an FB from the ear requires special competence and should therefore only be performed by specifically skilled professional staff.

## 1. Introduction

Foreign bodies (FBs) are the reason for 11% of consultations in the ENT emergency department [1]. The ear appears to be the most common site for FBs in the ENT area [1,2]. Patients with FBs in the ear may present with a variety of symptoms: hypoacusis, otalgia, otorrhagia, otorrhea, tinnitus, plugged ear, vertigo, nausea, taste disturbance, and cranial nerve palsy [1,3,4]. FBs can be classified according to insertion mode as incidentally or voluntarily [1,2]. They can vary widely in shape, size, and nature. Regarding the nature, FBs can be classified as organic or inorganic, animate or inanimate, metallic or nonmetallic, hygroscopic or non-hygroscopic, soft or hard, and regular or irregular [5]. FBs are usually located in the outer ear, rarely in the middle ear, and very rarely in the inner ear, causing perilymphatic fistula (PLF). PLF is defined as an abnormal communication between the fluid (perilymph)-filled space of the inner ear and the external surface of it, allowing perilymph to leak and causing specific disruptions in hearing, balance, or both. There are various causes: trauma, penetrating ear trauma, middle and inner ear diseases or surgery, barotrauma, or idiopathic. The treatment of PLF depends on the etiology and severity of symptoms. Treatment options include conservative treatment—such as avoiding activities that increase inner ear or intracranial pressure—administration of intratympanic steroids, and surgical intervention involving sealing of the fistula [6].

FBs need to be removed immediately. Specific surgical skills and techniques are required to minimize complications, including canal laceration, tympanic membrane perforation, displacement or retention of an FB in the inner ear, ossicular chain disruption, infection, perilymphatic fistula, and permanent sensorineural hearing loss [1,2]. General anesthesia is necessary in 8.6% to 30% of cases [1,2,3]. Here, we present a very rare case of inner ear injury resulting in PLF due to an FB after contact with a fish. Below, we discuss the challenges of managing such injuries during pregnancy, when diagnostic and anesthetic options may be limited.

## 2. Case Report

**Figure 1:**
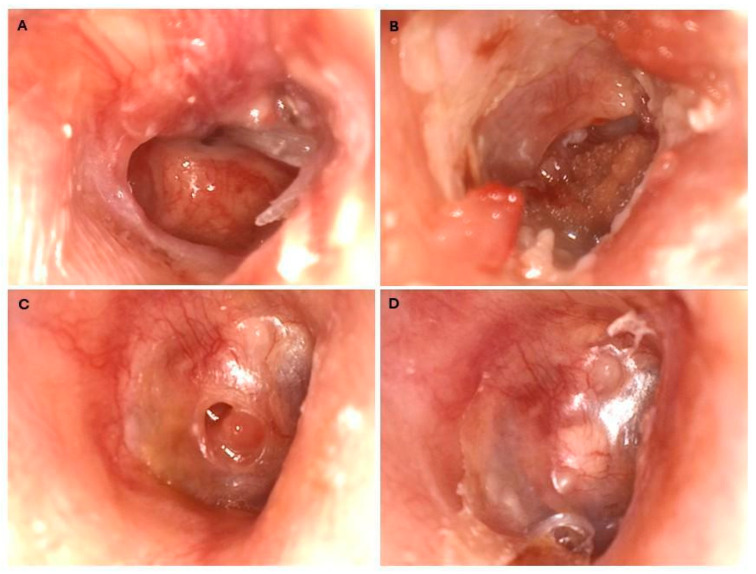
Tympanic membrane video otoscopic view: (**A**) before FB extraction; (**B**) three weeks after FB extraction; (**C**) six weeks after FB extraction; (**D**) after CI implantation and tympanoplasty.

A 33-year-old healthy female physiotherapist, with no history of previous illnesses, medication use, allergies, exposure to noxious substances, or relevant family history, was on holiday in Puerto Rico during the seventh week of her pregnancy. While swimming near the shore, a wave washed a fish toward her. She immediately noted pain in her ear, lost orientation, and fainted. She was rescued from drowning and taken to the beach. She suffered from nausea, vomiting, otalgia, otorrhea, and hearing loss ipsilaterally. On the same day, she visited a local doctor and received topical ciprofloxacin. As the condition had not improved, three days after she visited an ENT specialist in the local hospital. There, she rejected the proposed options of FB removal with or without anesthesia because she had to fly home to Austria the next day. The patient presented at the local hospital in Austria. As the attending ENT doctor had completed his scuba diving course in this region and heard about fish-related outer injuries through a diving instructor, an organic origin of the FB was initially suspected. When checking in 6 days after the trauma at our ENT department, a tertiary academic medical center, the patient presented with vertigo and hearing loss. The clinical examination showed an intact mastoid, pinna, and ear canal. An ear video-otoscopy showed that the tympanic membrane was subtotally perforated. A thin, long stick was stuck in the middle ear with one end lying in the anterior ear canal wall and the other end in the posterior superior quadrant (Figure 1A). To avoid exposure to radiation because of the pregnancy, it was not possible to perform a temporal bone scan. An MRI scan, which can be performed during pregnancy without any concerns, was not performed, as it would not have provided any additional information. Also, due to dizziness, the patient was unable to lie still during the examination. The challenge was that it was unclear how deeply the FB had penetrated; both the middle ear and the inner ear could have been affected. There was also a risk of further penetration into the inner ear canal and an intense form of liquorrhoea, known as a ‘gusher’.

Further treatment depended on clinical examination and other non-radiological tests, which complicated the treatment planning process.

**Figure 2:**
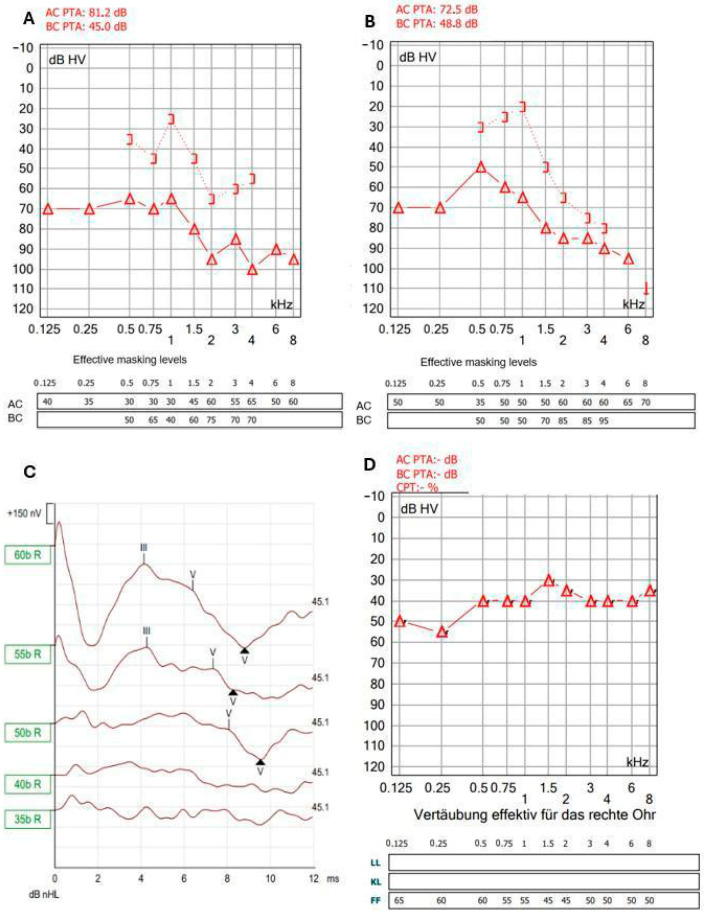
Pure-tone audiometry: (**A**) with FB; (**B**) after FB extraction; (**C**) four weeks after FB extraction; (**D**) six weeks after FB extraction.

The physical examination assessed the function of the middle and inner ear, including that of the facial nerve, which runs through the middle ear. The hearing tests showed the following results: Weber test (512 Hz) lateralized right, but Rinne test was negative. Pure-tone audiometry showed mixed hearing loss on the right side (Figure 2A).

Left-beating horizontal nystagmus grade II was registered under Frenzel glasses. Facial nerve function was normal.

**Figure 3:**
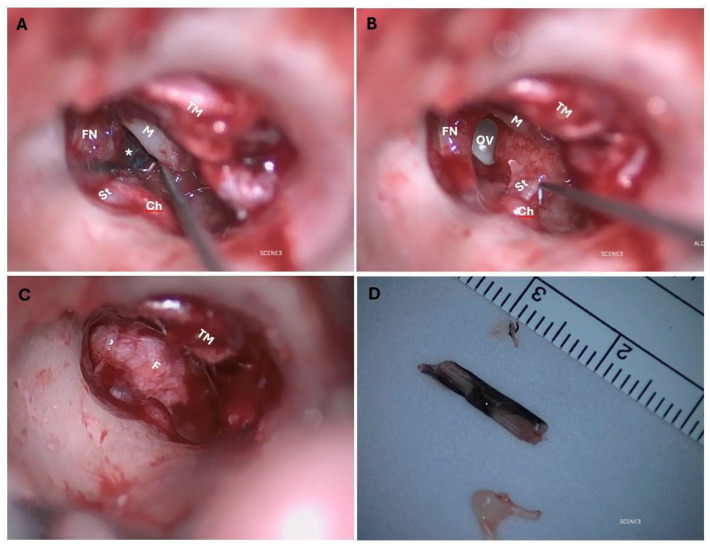
Intraoperative images: (**A**) before FB extraction; (**B**) after FB extraction; (**C**) after inner ear closure; (**D**) the extracted FB; TM: tympanic membrane; M: malleus; St: stapes; Ch: chorda tympani; FN: facial nerve; OV: oval window. The * indicates the location of the FB before its extraction.

The appropriate antibiotic for prophylaxis was selected based on its ability to cross the blood–brain barrier. Intravenous antibiotic (Ceftriaxon, MiP Pharma, Innsbruck, Austria) was administered, and explorative tympanoscopy with removal of the FB was performed under general anesthesia. A transcanal approach was chosen; the FB was located anterior to the auditory ossicles, entering the oval window and narrowly missing the facial nerve. The inner ear was devoid of fluid (Figure 3A–C).

**Figure 4:**
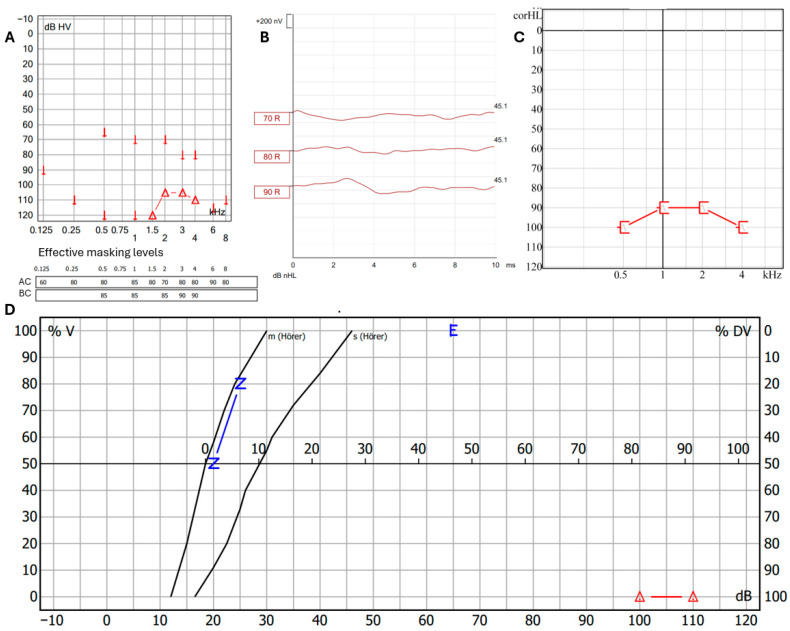
Hearing tests 6 weeks after FB extraction: (**A**) pure-tone audiometry; (**B**) ABR; (**C**) ASSR; (**D**) Freiburger speech audiometry.

Incudomalleolar and incudostapedial joints were separated; the stapes suprastructure was free-floating in the middle ear and therefore removed. Removing the superstructure of the stapes reduces the efficiency of sound transmission even after reconstruction. The facial nerve and chorda tympani were preserved. The middle ear was cleaned with saline solution, and the oval window niche was sealed with fascia to preserve the inner ear. Because of the infection risk, the tympanic membrane was not closed primarily. The FB was 15 mm long, 3 mm wide, 1.5 mm thick and appeared to be a part of a fish (Figure 3D). It was placed in a 96% alcohol solution and sent to the laboratory of the BOKU University Vienna, Austria. The genetic analysis showed that the FB belonged to a species within the genus *Hemiramphus*, a fish that is indexed as harmless to humans by FishBase [7]. Antibiotic therapy with ceftriaxone was administered for 8 days after surgery, and the patient was discharged on the fourth day after surgery. The patient rested as much as possible after surgery for the subsequent 2 weeks. The patient’s hearing and the condition of the eardrum were monitored after surgery. The prognosis was unclear. The tympanic membrane showed a tendency towards spontaneous closure (Figure 1B–D). Tone audiometry showed progressive hearing loss at high frequencies, while ABR remained stable. (Figure 2B,C). Six weeks after surgery, the patient presented back at the outpatient clinic because of deafness in the operated ear. The hearing test confirmed this suspicion using PTA, ABR, ASSR, and Freiburger Speech Audiometry (Figure 4A–D).

One month later, the hearing test battery was repeated with the same outcome and it was decided to perform cochlear implantation to restore hearing. Because sequelae of inner ear trauma, such as cochlear ossification or obliteration, may impact the outcome of cochlear implantation, we opted to perform the procedure during pregnancy. A SONNET 2 PIN cochlear implant with a FLEXSOFT electrode array (MED-EL, Innsbruck, Austria) was implanted. The patient was provided psychological support. Two months after implantation, the hearing was on a higher level, which was a positive development (Figure 2D). The Freiburg speech recognition test in quiet for maximal recognition level of numbers at 65 dB and maximal recognition level of maximum of monosyllabic words at 65 dB [8] were 100% and 35% 2 months after implantation and 100% and 65% 7 months after implantation. The patient gave birth to a healthy baby on time.

## 3. Fish Identification

DNA isolation was carried out using an SDS buffer system with a cleanup using silica membrane columns as described earlier [9]. A ~0.3 cm² piece of the material was digested in 300 µL Lysis buffer with 13 µL Proteinase K (10 mg/mL). DNA was eluted three times using 50 µL Tris buffer (10 mM, pH 8) and checked by gel electrophoresis. One microliter of the second elution was used in a 10 µL PCR reaction (Tm = 52 °C, 35 cycles) using QIAGEN Multiplex PCR Mastermix (Qiagen, Hilden, Germany) and 2 µL each of 1 mM forward and reverse primers. The PCR and following procedures were also replicated with 1:10 diluted DNA. We used different primer pairs to identify possible sample origins. Universal primers can sometimes suppress target amplification, whereas specific primers may over-amplify the target sequence, introducing bias. We therefore used three markers: one fish-specific mitochondrial marker covering parts of the tRNA-Pro gene and the adjacent D-loop region [10], a vertebrate-specific Cytochrome B marker (CypCytB_16026: GAAGAACCACCGTTGTWITTCAACTA, CCTCAGAAGGAYATYTGICCYCAIGG), and a universal primer pair for COI [11]. Primers were elongated by an Illumina-specific motif that can be used for library construction in a PCR. The PCR product was purified using magnetic beads [9], and a second PCR was performed to incorporate the Illumina adapter motifs, the index, and the sequencing primer recognition sequence in the amplicons. The resulting products were sequenced at the LMU Sequencing center in Munich on an Illumina MiSeq platform (Illumina, San Diego, CA, USA). Sequences containing the expected primer sequences at both ends were retained using cutadapt v4.8 (Freeware, Version 4.8, National Bioinformatics Infrastructure, Gothenburg, Sweden) [12] and fed into the DADA2 pipeline [13] for capturing Amplicon Sequence Variants (ASVs). The output was then compared to GenBank [14] and BOLD [15].

Results: The run resulted in 3000 raw reads for BF/HCO2198, 2868 reads for CypCytB, and 4844 reads for Bc33, when combining output for the undiluted and diluted DNA samples. After filtering and processing, the fish-specific primer pair Bc33 produced a single ASV with a total of 3882 reads. This showed the most similarity to whole mitochondrial sequences of *Hemiramphus balao* (OP056959.1, 84.58% identity) and *H. brasiliensis* (NC_088002.1, 84.38% identity). The primer pair CypCytB yielded six ASVs, five of which matched Homo sapiens (79%, 901 reads). The second most frequent ASV (21%, 235 reads) matched *H. balao* and *H. brasiliensis* sequences (91.32% identity) consistent with the references identified using Bc33. The COI amplification produced 15 ASVs. One matched human mitochondrial DNA (42%), and twelve matched human nuclear sequences or pseudogenes (0.8–13%). Two ASVs matched non-human sequences: one (Darwinula stevensoni, 21 reads, 100% identity in BOLD), and one (*Hemiramphus balao* and *H. brasiliensis*, 13 reads, 100% identity).

**Figure 5:**
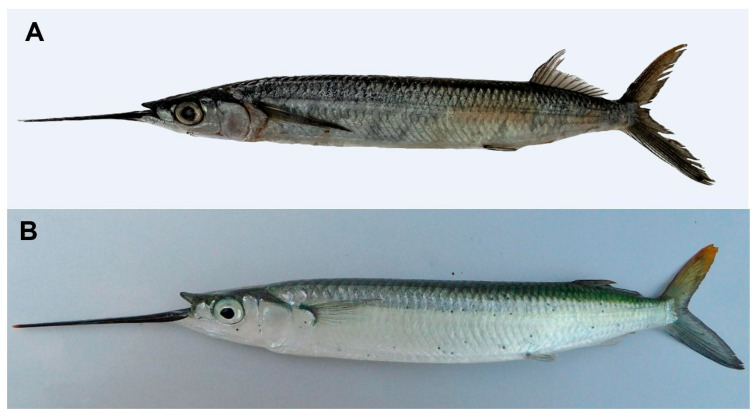
Images of two *Hemiramphus* species investigated in relation to the patient’s ear trauma: (**A**) *Hemiramphus balao*, photographed in Rio de Janeiro, Brazil, by Caio Henrique G. Cutrim (from FishBase [7]); (**B**) *Hemiramphus brasiliensis* (approx. 35 cm), photographed in Florida, USA, by Theo Modder (from FishBase [7]).

The detected contaminating sequences were mostly similar to human DNA, as expected, and in one case to a crustacean, likely introduced through sample contamination. Across all three markers, the fish-specific sequences were consistently identified as belonging to the genus *Hemiramphus*, most likely to the species *H. balao* or *H. brasiliensis* (Figure 5). Both species share similar mitochondrial haplotypes and cannot be distinguished based on the available data.

In conclusion, the analysis identified the fish DNA as belonging to the genus *Hemiramphus.* The sequences suggest a likely assignment to *H. balao* or *H. brasiliensis*; this cannot be confirmed due to the high similarity among mitochondrial haplotypes within the genus.

## 4. Recommendations for Managing Foreign Bodies in the Ear

The removal of an FB from the ear should be conducted promptly. The preparation involves identifying FB localization in the outer, middle, or inner ear. A detailed medical history should be documented, focusing on the duration of the FB presence in the ear, and vestibular symptoms. Key ear-related symptoms are otalgia, otorrhea, otorrhagia, hypoacusis, taste disturbance, and tinnitus, alongside vestibular dysfunction symptoms, such as vertigo, nausea, vomiting, intolerance to head motion, and postural instability. Clinical examination for FB in the ear should involve ear microscopy, hearing tests, and an assessment of the facial nerve function and testing for nystagmus. Ear microscopy identifies the type and the depth of the FB in the ear. Hearing tests help to ascertain if and which parts of the auditory pathway have been damaged. Conductive hearing loss is due to problems with the sound-conducting system (outer and/or middle ear), while sensorineural hearing loss is due to problems with the sound-transducing system (inner ear, the auditory nerve, or its central pathways). Occasionally, individuals are affected by mixed hearing loss, which is a combination of the two types of hearing loss. The simple screening tests with tuning forks can be used in primary care, postoperative settings, or to confirm audiological findings to distinguish sensorineural and conductive hearing loss. The Weber test is often combined with the Rinne test to detect the location and nature of the hearing loss. The Rinne test compares air and bone conduction to detect conductive hearing loss (AC > BC is normal). The Weber test assesses sound lateralization: it lateralizes to the affected ear in conductive loss and to the opposite ear in sensorineural loss [16]. Further audiological examinations—including pure-tone audiometry, speech audiometry, auditory steady-state response (ASSR), and auditory brainstem response (ABR)—should be performed to confirm and quantify the degree and type of hearing loss. Testing for nystagmus should be performed in cases of suspected vestibular involvement. If present, the nystagmus should be graded according to severity. Grade I nystagmus only appears when looking in the direction of the fast component, Grade II appears when looking straight as well, and Grade III is present in all directions of gaze, including toward the slow component.

When evaluating a suspected FB in the ear, differential diagnoses to consider include cerumen impaction, otitis externa, traumatic injury, cholesteatoma, and neoplastic lesions.

The type of FB, its localization, the patient’s age, willingness to cooperate, and previous removal attempts and symptoms are the factors that determine if the FB can be removed under local or general anesthesia. Easy visible and graspable FBs with a smooth and soft surface, located distally, without bleeding, without an otorrhea infection, and without any other symptoms in cooperative patients can be removed with adequate equipment by a physician or ENT specialist under local anesthesia. Medially impacted FBs in uncooperative patients with sensorineural or combined hearing loss, facial nerve palsy, or vestibular symptoms, after multiple attempts, should be removed under general anesthesia. If the temporal membrane is suspected to be perforated with dislocation of the FB behind TM, a CT scan should also be performed [5,17]. In addition to FB removal, further treatment may focus on hearing restoration. This can include tympanic membrane and ossicular chain reconstruction, sealing of a perilymphatic fistula, use of hearing aids, or cochlear implantation.

## 5. Discussion

While FBs in the external ear are very common in medical practice, FBs in the inner ear are extremely rare. Only a few cases have been reported: a migrated dental needle [18], nail from pneumatic nail gun [19], twig [4], metal pen tip [20], and yucca plant leaf spine [21]. To the best of our knowledge, this is the first case of animal-induced inner ear trauma with FB resulting in PLF. There was only one case reported with damage to the middle ear in which a fish was involved. Bijoor et al. described the case of an 11-year-old boy injured when swimming in the sea during a holiday in Oman. The authors hypothesized that a needlefish perforated the tympanic membrane. They found the 2.5–5 cm long FB in the middle ear, partially located in the Eustachian tube. Histological examination confirmed that the FB were bones. FB removal and myringoplasty were performed [22]. The FB pictures from this article seem to be very similar to our findings. In our case, we could confirm our suspicions thanks to cooperation with veterinary colleagues.

If vertigo, tinnitus, or nystagmus is present, concurrent fractures of the foot plate have to be considered. Clear diagnosis and proper therapy by skilled professionals are required. Silverstein et al. advised middle ear exploration within 24 hours of injury, warning that non-detection of inner ear damage could lead to severe permanent sensorineural hearing loss [23]. According to the literature, between 16.05% and 35.15% of ENT patients were referred from other clinical units to tertiary centers, the majority of them after an attempt to remove FB [1,2]. Previous attempts to remove FB were associated with a significantly higher rate of complications [2,17,24]. In our case, colleagues at the primary hospital correctly determined the risk of inner ear trauma, and no attempts were made. Transportation to the tertiary center may result in a delay in treatment. In the case of our patient, it took 6 days until the FB was removed. In other reported cases, the FB in the inner ear remained 16 [4] and 14 days [20] before extraction. To diagnose damage to the inner ear and determine the surgical strategy, a high-resolution CT scan of the temporal bone is useful. Due to our patient’s pregnancy, no CT scan was performed and the FB was removed under general anesthesia. The localization of the FB medial to the tympanic membrane is one of the criteria suggested by Ponnuvelu to decide to remove the FB under general anesthesia [5]. If trauma-related PLF is suspected, explorative tympanoscopy with connective tissue closure should be applied. Bogaerts et al. describe 13 cases of stapedio-vestibular luxation due to penetrating trauma. He reported that a PLF typically heals on its own if the lesion is small [25]. Although the connective tissue showed signs of spontaneous closure, our patient’s PLF was large, and the FB seemed to prevent closure. The prognosis for hearing in patients with PLF is generally poor. Some patients experienced improvements in bone conduction as a result of early surgical intervention, even if their hearing was already impaired prior to surgery. At first, our patient retained partial hearing, but over time she experienced complete hearing loss and cochlear implantation was required. Vertigo has a better prognosis than hearing loss; it usually subsides a few days after surgery [25]. PLF is a pathway of communication between the middle ear and the intracranial space, and recurrent meningitis has been seen as a complication of an unrepaired oval window rupture. In our case, to prevent complications from the contaminated FB, antibiotics were given and the primary tympanic closure was delayed.

## 6. Conclusions

The patient presented in this report had an unusual mechanism of inner ear trauma. The FB, identified as part of a fish, was genetically analyzed and determined to be most likely from the fish species *Hemiramphus balao* or *H. brasiliensis* or a closely related species, which is indexed as harmless to humans in FishBase. The injury did not affect the facial nerve. As severe penetration of an FB into the middle and inner ear can lead to serious complications, removal of an FB from the ear requires special competence and should therefore only be performed by specifically skilled professional staff. Further observation of the patient is required, as delayed effects may occur and additional intervention might be required. We recommend a 3-month observation after injury.

## Data Availability

No new data were created or analyzed in this study. Data sharing is not applicable to this article. Images of fish species were reproduced from FishBase (www.fishbase.org (accessed on 6 November 2025)) with proper attribution and in accordance with the site’s usage terms. Permission to reproduce these images was granted directly by the original photographers, Theo Modder and Caio Henrique G. Cutrim, via personal email correspondence.

## Abbreviations

The following abbreviations are used in this manuscript:

FB	Foreign body
PLF	Perilymphatic fistula
CI	Cochlear Implantation
